# Aldosterone Induces the Proliferation of Renal Tubular Epithelial Cells *In Vivo* but Not *In Vitro*

**DOI:** 10.1155/2021/9943848

**Published:** 2021-07-26

**Authors:** Juan Hao, Lingjin Liu, Ziqian Liu, Gege Chen, Yunzhao Xiong, Xiangting Wang, Xuelian Ma, Qingyou Xu

**Affiliations:** ^1^Graduate School, Hebei University of Chinese Medicine, Shijiazhuang, China; ^2^Hebei Key Laboratory of Integrative Medicine on Liver-Kidney Patterns, Hebei University of Chinese Medicine, Shijiazhuang, China; ^3^Department of Internal Medicine, Hebei University of Chinese Medicine, Shijiazhuang, China

## Abstract

**Objective:**

To investigate the proliferation effect of aldosterone on renal tubular epithelial cells *in vivo* and *in vitro*.

**Methods:**

Thirty-two male C57BL/6J mice (20–22 g) were divided randomly into four groups: sham, unilateral nephrectomy (UN), unilateral nephrectomy plus aldosterone infusion (UA), and UA plus eplerenone (UAE). The kidneys were removed 6 weeks after treatment. Expression of proliferating cell nuclear antigen (PCNA) was detected by immunohistochemistry and western blotting. Human kidney proximal tubular epithelial (HK2) and mouse distal convoluted tubule (mDCT) cell lines were stimulated by aldosterone (0, 10^−9^, 10^−8^, 10^−7^, and 10^−6^ mol/L) *in vitro*. Cells were collected after 3, 6, 12, 24, 36, and 48 h, and proliferation of each group detected by western blotting, flow cytometry, live imaging, and the MTT assay. In addition, mDCT cells were costimulated with a medium containing a final concentration of 161 mmol/L Na^+^ and different concentrations of aldosterone, and the number of cells and cellular DNA content was measured by the MTT assay and flow cytometry.

**Results:**

Aldosterone could induce a significant increase in the number of PCNA-positive cells in mouse kidneys accompanied by increased deposition of collagen fibers. Eplerenone could inhibit aldosterone-induced cell proliferation and collagen deposition. HK2 cells and mDCT cells administered different concentrations, and different times of aldosterone stimulation failed to cause cell proliferation, and costimulation of aldosterone and salt did not cause proliferation changes in mDCT cells.

**Conclusions:**

Aldosterone perfusion can induce proliferation of mouse kidney cells *in vivo*, and eplerenone can inhibit this change, but aldosterone stimulates HK2 cells and mDCT *in vitro* without causing their proliferation.

## 1. Introduction

Fibrosis is the pathologic change in chronic kidney disease, and the latter progresses to end-stage renal failure. The main pathologic features of fibrosis are organ atrophy, excessive deposition of the extracellular matrix (ECM), and cell proliferation [1].

Excessive deposition of the ECM is the most obvious marker of renal interstitial fibrosis. Cell proliferation usually occurs in the early stages of fibrosis and persists in ECM accumulation and glomerulosclerosis [2]. Ruster and colleague showed that more than one-third of fibroblasts in renal interstitial fibrosis are derived from renal tubular epithelial cells (RTECs) [3]. Inhibition of cell proliferation can reduce ECM accumulation. Conversely, stimulation of cell proliferation leads to increased deposition of the ECM. Previously, we showed that 10 days after unilateral ureteral obstruction (UUO), high expression of proliferating cell nuclear antigen (PCNA) appeared in the RTECs of the contralateral kidney accompanied by ECM deposition [4]. Those data suggested that RTEC proliferation may be involved in the development of renal fibrosis.

Aldosterone is the main active substance of mineralocorticoids and mediates the homeostasis of salt and water through interaction with mineralocorticoid receptors (MRs) which are expressed in renal epithelial cells [5, 6]. The role of aldosterone in chronic kidney disease and the protective effect of mineralocorticoid receptor blockers (MRB) have received attention in recent years. Aldosterone stimulates MR activation to participate in the formation of fibrosis through cell proliferation (including glomerular mesangial cells [7], RTECs [4], lung macrophages [8], and cardiac fibroblasts [9]), phenotypic transformation, and others [10–13]. This pathological change can be blocked by MRB effectively. Previously, we showed that the RTECs and interstitial cells of the contralateral kidney can proliferate after 10 days of UUO [4]. Although there have been reports of the direct proliferation effect of aldosterone on interstitial *in vitro* [14–16], reports of the direct effect of aldosterone on RTECs are lacking. Can aldosterone stimulate the renal tubular cell proliferation in animals and especially in vitro directly? Can MRB effectively inhibit the aldosterone-induced renal tubular epithelial cell proliferation?

To further explore the mechanism of action of aldosterone on RTEC proliferation, mice were infused with aldosterone *in vivo*, and RTECs were stimulated by different concentrations of aldosterone *in vitro*. Our purpose was to explore the role of aldosterone in hypertrophy and fibrosis of the contralateral kidney early after UUO, and how eplerenone intervenes in this process.

## 2. Materials and Methods

### 2.1. Materials

Aldosterone was purchased from Cayman Chemicals (Ann Arbor, MI, USA). Fetal bovine serum (FBS) and penicillin–streptomycin were obtained from Gibco (Grand Island, NY, USA). Dimethyl sulfoxide, 3-(4,5-dimethylthiazol-2-yl)-2,5-diphenyltetrazolium bromide (MTT), Cell Cycle Detection Kit, and solutions used for trypsin digestion were purchased from Beijing Solarbio Science & Technology (Beijing, China). PCNA was obtained from Proteintech (Wuhan, China).

### 2.2. Design of Animal Experiments

The study protocol was approved by the Committee on the Ethics of Animal Experiments of Hebei University of Chinese Medicine (Hebei, China). Experiments were undertaken in accordance with the guidelines set by the US National Institutes of Health (Bethesda, MD, USA).

Thirty-two male C57BL/6J mice (8 weeks; 20–22 g; HFK Laboratory Animals, Beijing, China) were used to test the proliferation effect of aldosterone (as a growth-promoting substance) on RTECs. Mice were maintained with standard mouse chow and tap water at 22°C under a 12 h light–dark cycle.

Mice were divided into four groups of eight randomly: vehicle (sham), unilateral nephrectomy (UN), unilateral nephrectomy + aldosterone infusion (UA), and UA + eplerenone (UAE).

For the UA group, mice underwent left unilateral nephrectomy under anesthesia (isoflurane). After 5 days of recovery from surgery, an osmotic minipump (1002 series; Alzet, Cupertino, CA, USA) was implanted (s.c.) to infuse aldosterone (0.75 *μ*g/h) [17] for 6 weeks.

For the UAE group, mice that had undergone unilateral nephrectomy + aldosterone infusion were given 100 mg·kg^−1^d^−1^ of eplerenone (Pfizer, New York, NY, USA), a specific blocker of MRs, in their feed.

Mice were allowed free access to food and drinking water. Kidney samples were harvested at the end of week 6. After decapitation, the right kidney was removed, weighed, fixed in 10% formalin, and embedded in paraffin for histology.

### 2.3. Cell Culture

Human kidney proximal tubular epithelial (HK2) cells were obtained from Procell Life Science & Technology (Wuhan, China). Mouse distal convoluted tubule (mDCT) cells were obtained from Professor Tatsuo Shimosawa (International University of Health and Welfare, Tokyo, Japan). Both types of cells were grown in an atmosphere of 95% air and 5% CO_2_ at 37°C. Cells were maintained in Dulbecco's modified Eagle's medium (DMEM)/F12 medium (consisting of DMEM, 10% FBS, 10,000 units/mL of penicillin, and 10,000 *μ*g/mL of streptomycin) in a humidified incubator. A high-Na^+^ medium (145.3 mmol·L^−1^) contained additional sodium chloride, and the final concentration of Na^+^ was 161 mmol·L^−1^. Cells were plated at 1 × 10^5^ cells/mL in 96-well plates or flasks. On day 1, cells underwent trypsinization by incubation in a solution of 0.25% trypsin and 0.02% ethylenediamine tetra-acetic acid in phosphate-buffered saline (PBS) for 1–2 min at 37°C. One milliliter aliquots of a cell suspension were distributed to a 96-well plate, or 6 mL aliquots of cell suspension were distributed to flasks. On day 2, the culture medium was replaced with serum-free medium for 24 h to render the cells quiescent. Subsequently, cells were incubated in serum medium treated with aldosterone (0, 10^−9^, 10^−8^, 10^−7^, and 10^−6^ mol/L) diluted in ethanol for 3, 6, 12, 24, 36, or 48 h. Cells were harvested at different times.

### 2.4. Staining

Staining (Sirius Red, Masson) was carried out using standard procedures. The kidneys were fixed in 10% neutral buffered formalin and embedded in paraffin. The presence of interstitial fibrosis in kidney sections was assessed by staining.

### 2.5. Immunohistochemistry

The kidneys were fixed overnight in 4% paraformaldehyde and embedded in paraffin. Six micrometer sections were cut, dewaxed, and dehydrated in a graded series of alcohol solutions. Antigens were retrieved in a citric acid solution (pH 6.0, 95°C–100°C) for 20 min. Next, sections were placed in an atmosphere of 3% H_2_O_2_ for 20 min and then incubated in 10% goat serum for 30 min. Sections were probed with primary antibodies against PCNA (1 : 300 dilution; catalog number: 0086617; Proteintech) overnight at 4°C. Then, sections were washed and incubated for 15 min with biotinylated secondary antibodies. Upon completion of incubation, the signal was amplified by formation of an avidin biotin complex and was developed by counterstaining with diaminobenzidine and hematoxylin.

### 2.6. Western Blotting

Cells were lysed and sonicated in cold PBS. Then, 30 *μ*g of protein from the whole-cell preparation was denatured in boiling water for 15 min, separated by sodium dodecyl sulfate–polyacrylamide gel electrophoresis, and transferred onto polyvinylidene difluoride (PVDF) membranes. As an index of their proliferation activity, mDCT cells and HK2 cells expressing PCNA were assessed. Immunoblotting was done with primary antibodies against PCNA (1 : 1000 dilution), followed by the addition of appropriate fluorescence-labeled secondary antibodies.

### 2.7. Cell Viability/Proliferation according to the MTT Assay

The number of viable cells was determined by incubating cell cultures with MTT (5 mg/mL) for 4 h in a humidified atmosphere of 5% CO_2_. Then, 150 *μ*L of dimethyl sulfoxide was added at room temperature for 10 min. Finally, absorbance was measured at 570 nm using a microplate reader (Molecular Devices, Silicon Valley, CA, USA).

### 2.8. Analysis of the Cell Cycle

Cells were seeded in a six-well plate and administered aldosterone as described in Section 3.2. After treatment, cells were collected and washed with PBS. Subsequently, cells were fixed in ice-cold 70% ethanol overnight and reacted with RNase A at 37°C for 30 min. Finally, propidium iodide (0.01 mg/mL) was added and incubation allowed for 30 min at 4°C. DNA content was detected by flow cytometry. Data were analyzed by Cell Quest™ (Beckman Coulter, Fullerton, CA, USA). Cells were counted (10,000–20,000) and analyzed as the percentage in G1, S, or G2 phases.

### 2.9. Imaging of Live Cells

The prepared cell suspension was added to a 96-well plate at 100 *μ*L per well. This action was followed by placement in Incucyte® S3 (Essen Bio Science, Ann Arbor, MI, USA). A visual field with a relatively average number of cells was selected at an interval of 1 h. The degree of cell fusion and the number of cells were observed 48 h after aldosterone stimulation.

### 2.10. Statistical Analyses

Statistical analyses were carried out using SPSS 24.0 (IBM, Armonk, NY, USA). Results are the mean ± SD. Different groups were compared by the Student's *t*-test. Differences among groups were compared by one-way analysis of variance (ANOVA), followed by the least significant difference/Student–Newman–Keuls test. Dose-dependent differences were compared by one-way ANOVA, followed by Tukey's honestly significant difference test. *P* < 0.05 (two-tailed) was considered significant.

## 3. Results

### 3.1. Changes in Interstitial Fibrosis in Mouse Kidneys Induced by Aldosterone Perfusion Can Be Reversed by Eplerenone

**Figure 1:**
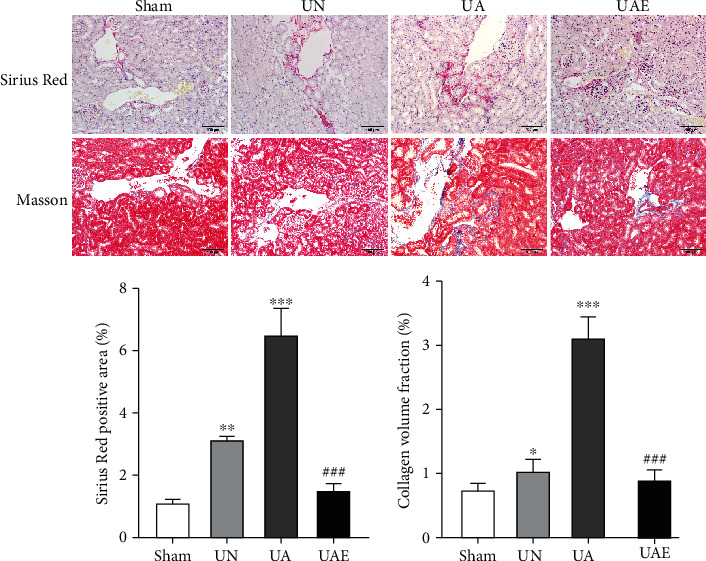
Eplerenone attenuates renal fibrosis in aldosterone-infused mice. Staining (Sirius Red, Masson) was used to evaluate the degree of renal fibrosis. In the UA group, collagen fibers were deposited around blood vessels. *N* = 3. Values are the mean ± SD, ^∗^*P* < 0.05 vs. sham, ^∗∗^*P* < 0.01 vs. sham, ^∗∗∗^*P* < 0.001 vs. sham; ^#^*P* < 0.05 vs. UA, ^##^*P* < 0.05 vs. UA, ^###^*P* < 0.05 vs. UA. UN group: unilateral nephrectomy for 6 weeks; UA group: unilateral nephrectomy + aldosterone perfusion. 5 days after surgery, an osmotic minipump was implanted (s.c.) to infuse aldosterone (0.75 *μ*g·h^−1^) for 6 weeks. UAE group: UA mice were given eplerenone (100 mg·kg^−1^d^−1^).

To ascertain the effect of aldosterone perfusion on renal fibrosis after unilateral nephrectomy, we undertook staining (Sirius Red, Masson) on kidney tissue. In the UA group, Many collagen fibers are deposited around the blood vessels. Expression of collagen in the UA group was enhanced significantly, and expression of collagen in the UN group was slightly enhanced than that in the sham group (Figure 1).

### 3.2. Aldosterone Induced PCNA Expression *In Vivo* and *In Vitro*

**Figure 2:**
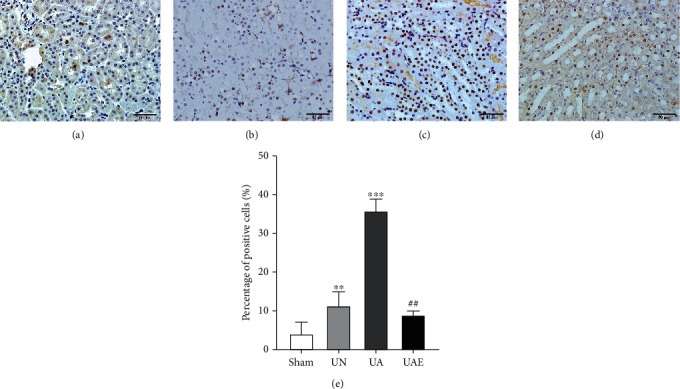
Eplerenone inhibits the expression of PCNA in aldosterone-fusion mice. Immunohistochemical staining for PCNA in kidney sections from different groups. (a) Sham group. (b) UN group: the left kidney of the mouse was removed, and the right kidney was collected 6 weeks later. (c) UA group: aldosterone (0.75 *μ*g·h^−1^) was administered for 6 weeks. (d) UAE group: eplerenone was given to the UA group *via* diet at 1.25 g·kg^−1^ diet (equal to 100 mg·kg^−1^d^−1^) for 6 weeks, and other groups of mice were fed regular chow. (e) Quantification of PCNA. Data are the mean ± SD (*n* = 3). ^∗^*P* < 0.05 vs. sham group, ^∗∗^*P* < 0.01 vs. sham group, ^∗∗∗^*P* < 0.001 vs. sham group; ^#^*P* < 0.05 vs. UA group, ^##^*P* < 0.01 vs. UA group, ^###^*P* < 0.001 vs. UA group. Original magnification, ×400. PCNA: proliferating cell nuclear antigen; UA group: unilateral nephrectomy + aldosterone perfusion; UN group: unilateral nephrectomy; UAE group: UA + eplerenone.

We wished to detect the proliferation effect of aldosterone on RTECs *in vivo*, so we used immunohistochemistry to measure PCNA expression. After selection of three fields randomly, the number of PCNA-positive cells per tubule was counted using ImageJ (US National Institutes of Health). The percentage of PCNA-positive cells to the total number of cells was 3.89 ± 3.13% in the sham group, 10.98 ± 3.74% in the UN group, 35.37 ± 3.24% in the UA group, and 8.52 ± 1.46% in the UAE group. The number of positive cells in the UA group was increased significantly compared with that in the sham group and UN group (*P* < 0.01), but eplerenone could reduce PCNA expression (*P* < 0.01) (Figure 2).

**Figure 3:**
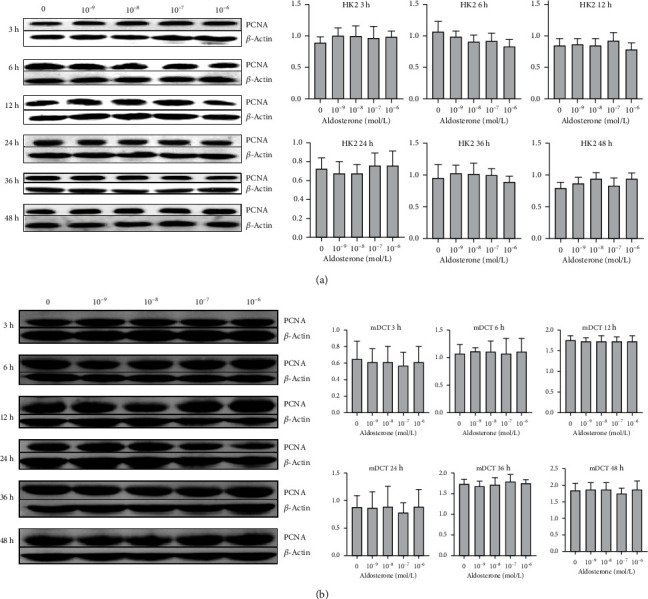
Effects of aldosterone on the protein expression of PCNA according to western blotting. (a) HK2 cells were treated with aldosterone (0, 10^−9^, 10^−8^, 10^−7^, and 10^−6^ mol/L) for 3, 6, 12, 24, 36, or 48 h. Total cellular protein was extracted, and 30 *μ*g of protein was loaded for western blotting of total PCNA and *β*-actin. (b) mDCT cells. Data are the mean ± SD (*n* = 3). HK2: human kidney proximal tubular epithelial; mDCT: mouse distal convoluted tubule epithelial; PCNA: proliferating cell nuclear antigen.

*In vitro*, we used HK2 cells and mDCT cells to measure the protein expression of PCNA. Administration of different concentrations of aldosterone had no effect on the PCNA expression in HK2 cells (Figure 3(a)) or mDCT cells (Figure 3(b)) compared with that elicited not using aldosterone (*P* > 0.05) at different stimulation times.

### 3.3. Effect of Aldosterone on HK2 Cells and mDCT Cells according to Dose, Time, and the Cell Cycle

**Figure 4:**
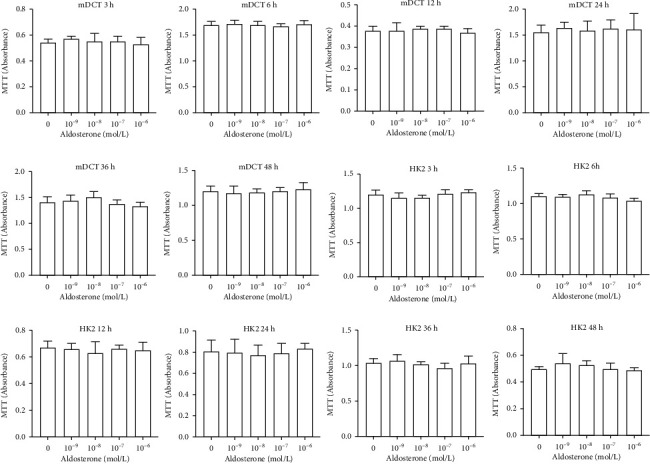
Dose and time effect of aldosterone on proliferation of mDCT cells and HK2 cells. Aldosterone-stimulated cell growth was measured using the MTT assay. The assay was carried out on mDCT cells and HK2 cells. Cells were divided into untreated (0 mol/L) or treated with different concentrations of aldosterone for 3, 6, 12, 24, 36, or 48 h. The absorbance measurement at 570 nm was recorded. The value represents the mean value of five separate experiments. Each average contains six auxiliary holes, and the results are plotted in a bar chart, mean ± SD (*n* = 6). mDCT: mouse distal convoluted tubule epithelial; HK2: human kidney proximal tubular epithelial.

We wished to ascertain the survival and growth of cells. The MTT assay was chosen to detect (indirectly) changes in the cell number at different times and concentrations. MTT assay demonstrated that in HK2 cell lines, compared with 0 mol/L of aldosterone, it did not proliferate stimulated by different aldosterone concentrations at different times; consistent with the results of HK2, mDCT did not proliferate (Figure 4). This result suggested that, under these test conditions, aldosterone did not stimulate the proliferation of HK2 cells.

**Figure 5:**
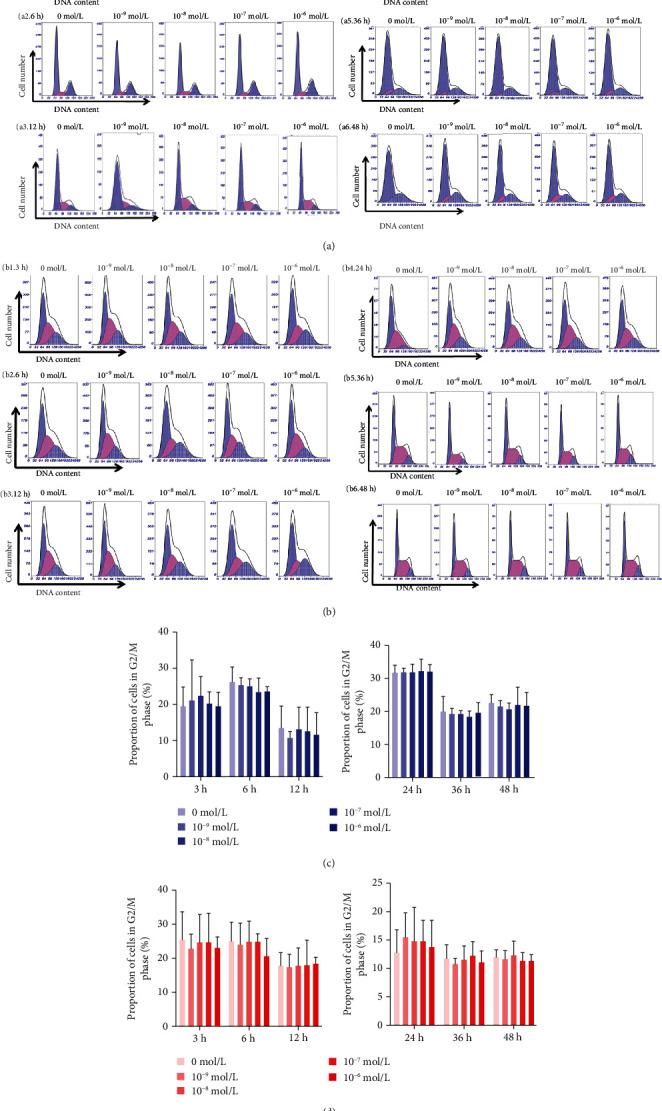
Effect of aldosterone on the cycle in HK2 cells (a) and mDCT cells (b). Effects of aldosterone (0, 10^−9^, 10^−8^, 10^−7^, and 10^−6^ mol/L) on the number and DNA content of HK2 cells for 3 (a1), 6 (a2), 12 (a3), 24 (a4), 36 (a5), or 48 (a6) hours. Effects of aldosterone on the number and DNA content of mDCT cells treated with aldosterone (0, 10^−9^, 10^−8^, 10^−7^, and 10^−6^ mol/L) for 3 (b1), 6 (b2), 12 (b3), 24 (b4), 36 (b5), or 48 (b6) hours. Percentage of cells in the G2/M phase cells is presented as a bar graph; each bar represents the mean ± SD (*n* = 3). (c) Proliferation cycle of HK2 cells. (d) mDCT cells. mDCT: mouse distal convoluted tubule epithelial; HK2: human kidney proximal tubular epithelial.

We wished to explore if aldosterone-induced cell proliferation was associated with cell-cycle arrest. We detected the distribution of cell-cycle phases in HK2 cells and mDCT cells using flow cytometry to analyze cellular DNA content. There was no significant increase in the percentage of HK2 cells or mDCT cells in the M/G2 phase after administration of different concentrations of aldosterone compared with that in the group not administered aldosterone (*P* > 0.05) (Figures 5(a) and 5(b)).

### 3.4. Combined Effect of Salt and Aldosterone on the Proliferation of mDCT Cells

**Figure 6:**
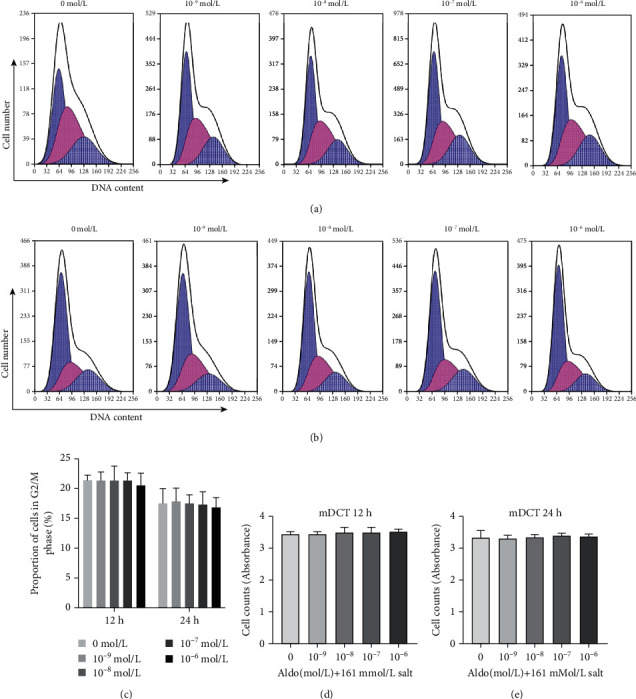
Synergistic effect of aldosterone and salt on proliferation of mDCT cells. mDCT cells were in a medium containing 161 mmol/L Na^+^. Cells were stimulated by different concentrations of aldosterone. Cell counting and flow cytometry were used to detect changes in the cell number and DNA content. (a) Change in DNA content after 12 h of stimulation and (b) change after 24 h of stimulation in mDCT cells. The result was presented in (c); values were given as the mean ± SD. (d) The cell counts of mDCT after were stimulated for 12 hours. (e) The cell counts of mDCT after were stimulated for 24 hours.

To ascertain if aldosterone and salt stimulate cell proliferation synergistically, we selected mDCT cells with more MR receptors to detect changes in cell number and DNA content during the cell cycle after 12 h and 24 h. Combined stimulation with high levels of salt and different concentrations of aldosterone did not cause significant changes in cell number or cellular DNA content compared with that in the group not administered aldosterone (*P* > 0.05) (Figure 6).

### 3.5. Live Imaging of HK2 Cells and mDCT Cells after Aldosterone Stimulation

**Figure 7:**
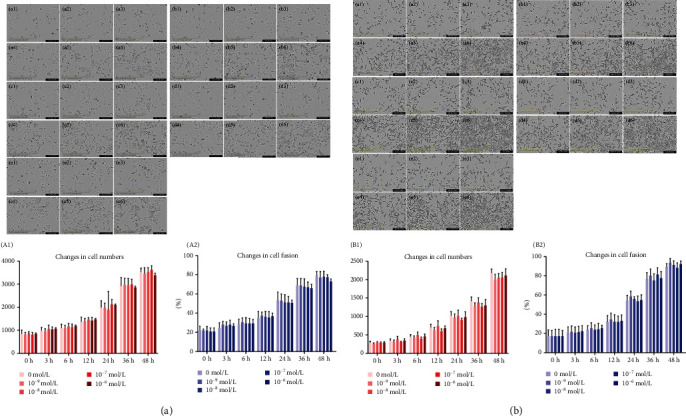
Growth of HK2 cells and mDCT cells observed by live imaging. Cells (1 × 10^5^ cells/mL) were plated into 96-well plates and stimulated by aldosterone (0, 10^−9^, 10^−8^, 10^−7^, and 10^−6^ mol/L) for 48 h. The time interval for recording images was 1 h. After 12 h, the cells began to adhere to the wall. We observed cell growth for 3, 6, 12, 24, 36, and 48 h. (a) HK2 cells. (b) mDCT cells. (A) Aldosterone concentration of 0 mol/L. (B) Aldosterone concentration of 10^−9^ mol/L. (C) Aldosterone concentration of 10^−8^ mol/L. (D) Aldosterone concentration of 10^−7^ mol/L. (E) Aldosterone concentration of 10^−6^ mol/L. 1, 2, 3, 4, 5, and 6 show cell growth at 3, 6, 12, 24, 36, and 48 h, respectively. We selected three replicate wells randomly, and the number of cells and the degree of fusion were calculated. Results are plotted as a bar chart, and values are the mean ± SD. (A1, B1) represent changes in the cell number, and (A2, B2) represent changes in cell fusion, compared with that in the group not given aldosterone.

We observed changes in the number of HK2 cells, mDCT cells, and their degree of fusion at different times and concentrations of aldosterone in real time. With increasing time, the degree of fusion and number of HK2 cell increased significantly (Figure 7(a), (A)–(E)). Compared with that in the group not administered aldosterone, the degree of cell fusion 36 h and 48 h after stimulation with aldosterone decreased slightly, but the difference was not significant (*P* > 0.05) (Figure 7(A1)). The number of cells stimulated by aldosterone (10^−6^ mol/L) decreased slightly at 36 h compared with that in the group not administered aldosterone, but the difference was not significant (*P* > 0.05) (Figure 7(A2)). Dynamic images of the number and fusion of mDCT cells are shown in Figure 7(b), (A)–(E), (B1), (B2).

## 4. Discussion

Aldosterone has a major role in electrolyte transport *via* MRs. It is synthesized in the endothelium, heart, vascular smooth muscle, and brain [17–19]. Previously, the renal distal tubule was considered to be the only target of aldosterone. Today, we know that nonepithelial tissues (e.g., heart, vessels, adipose tissue, and macrophages) are also potential targets for aldosterone [20]. In addition to ion transport, aldosterone can stimulate cell growth (e.g., glomerular mesangial cells [21], vascular smooth muscle cells [22], and cardiac fibroblasts [23]) in target tissues by activating signaling cascades rapidly. The stimulating effect of aldosterone on cells (hypertrophy or hyperplasia) is dependent upon the cell type.

If inflammation or damage occurs to renal tissue, aldosterone activates RTECs to secrete cytokines such as platelet-derived growth factor [24], epidermal growth factor [25] interleukin-4 [26], transforming growth factor [25], or tumor necrosis factor [27], which promote fibroblast proliferation directly in renal interstices. Conversely, aldosterone promotes cell-phenotype transformation and participates in the progression of renal fibrosis. Previously, we reported that in early UUO-induced injury, many tubular cells proliferate in the contralateral kidney accompanied by an increased aldosterone concentration in plasma and renal fibrosis [4]. The effect of aldosterone on the proliferation and differentiation of normal tubular cells has been demonstrated in young animals [28]. However, there is little evidence that an increased aldosterone level in plasma can induce the differentiation and proliferation of mature renal tubular cells. Therefore, we explored whether aldosterone can stimulate RTEC proliferation to induce renal fibrosis directly.

Aldosterone is the main active substance of mineralocorticoids. Some studies have shown that mineralocorticoids can promote the proliferation of glomeruli and tubular parenchymal cells in the acute phase, which accelerates chronic kidney disease. MR blockers have a protective effect against kidney damage. For example, in some models of acute kidney injury induced by ischemia–reperfusion in rats, spironolactone administered 24 h after ischemia was shown to reduce proteinuria and glomerular hypertrophy effectively [29]. In a model of unilateral nephrectomy with perfusion of salt and aldosterone, renal-tubular hypertrophy and excessive changes in glomerular hypercellularity have been observed. Eplerenone administration can alleviate those changes [30]. Previously, we reported that in early UUO-induced injury, RTEC proliferation in the contralateral kidney was reduced if the MR blocker eplerenone was given [4]. We speculated that proliferation of renal tubular cells was due to MRs.

To ascertain if cell proliferation is through MRs, mice who had undergone unilateral nephrectomy were given the MR agonist aldosterone. Mice who had undergone unilateral nephrectomy plus aldosterone perfusion were administered the MR blocker simultaneously. We measured the PCNA expression in kidney tissue. PCNA is a sensitive indicator of cell proliferation and plays an important role in DNA synthesis/repair and cell proliferation [31]. In mice perfused with aldosterone, the PCNA expression increased significantly compared with that in the sham group. Eplerenone administration could reduce the PCNA expression significantly. Consistent with the PCNA expression, the degree of renal fibrosis was more severe in UA-group mice, and fibrosis could be reversed by eplerenone.

HK2 cells and mDCT cells were selected to verify that aldosterone induces RTEC proliferation by activating MRs *in vitro*. We used four methods to verify aldosterone-induced proliferation in RTECs. The first method was using the MTT assay to indirectly verify the proliferation effect of aldosterone: a proliferation effect on RTECs was not observed. The second method was to measure the protein expression of PCNA. There was no significant difference under stimulation by different concentrations of aldosterone at different times. *In vitro*, aldosterone induced the proliferation of renal parenchymal cells (mainly glomerular mesangial cells [32]) but not RTECs. Third, we employed live imaging to detect the fusion and number of cells. With increasing culture time, the number of cells and degree of fusion increased but, under stimulation by different concentrations of aldosterone, the number and degree of fusion of cells did not change significantly. At an aldosterone concentration of 10^−6^ mol/L, the number of cells and degree of fusion decreased slightly, but not significantly. We speculate that this result may have been caused by the effect of aldosterone on cell injury. The fourth method was analyses of cell-cycle changes by flow cytometry. We tested the cycle changes of two cell lines under stimulation by different concentrations of aldosterone at different times. The G2/M phase in each group did not change significantly.

Studies have shown that aldosterone and salt have a synergistic effect on the development of salt-sensitive hypertension. Shibata and colleagues [33] found that, in a salt-sensitive rat model of adrenalectomy with aldosterone perfusion, high salt could induce Racl and aldosterone to interdependently cause MR overactivation and increase blood pressure. Some studies have shown that salt can also bind to MRs directly without reliance on the renin–angiotensin–aldosterone system (RAAS). For example, in salt-sensitive individuals, high salt intake can promote Na^+^ reabsorption independent of the RAAS by direct activation of MRs [34]. In rodent models of salt-sensitive hypertension, a high salt diet was shown to activate Rac1, increase blood pressure, and cause kidney injury despite suppressing aldosterone. Rac1 GTPase in rodent kidneys is also essential for salt-sensitive hypertension and works *via* an MR-dependent pathway [35]. In addition to Rac1, high intake of salt in the diet increased the serum level of glucocorticoid-induced kinase 1, a downstream effector of MRs [36], in salt-sensitive Dahl rats independent of aldosterone [37]. Combined stimulation with high levels of salt and different concentrations of aldosterone did not cause significant changes in cell number or cellular DNA content compared with that in the group not administered aldosterone in the present study.

## 5. Conclusions

Aldosterone administration increased the PCNA expression in RTECs *in vivo*, but aldosterone did not increase the PCNA expression in HK2 cells or mDCT cells. Other experimental methods did not demonstrate the proliferation effect of aldosterone on RTECs *in vitro*. Therefore, we speculate that obstructive nephropathy and other CKD have the activation of RAAS, which not only stimulates the secretion of aldosterone but also secretes a large amount of other vasoactive peptides and cytokines such as AngII, ET-1, and TGF-*β*1, which are involved in cell proliferation. The proliferation of renal tubular epithelial cells can be caused by different pathway. The activation of MR by aldosterone is one of the ways. Whether there are other ligands involved or some other substances that cooperate with aldosterone to cause MR activation, this phenomenon is expected, and researchers pay attention on it.

## Data Availability

If you need the basic data of this article, you can get in touch with the corresponding author by email (qingyouxu@sohu.com).
